# Patient tracking during treatment of children with cancer in India – An exploratory study

**DOI:** 10.1002/cnr2.1359

**Published:** 2021-02-23

**Authors:** S Ahuja, J Sharma, S Gupta, S Bakhshi, R Seth, A Singh, P Bagai, RS Arora

**Affiliations:** ^1^ Quality Care Research and Impact Cankids New Delhi India; ^2^ Division of Haematology/Oncology Hospital for Sick Children Toronto Canada; ^3^ Department of Medical Oncology All India Institute of Medical Sciences New Delhi India; ^4^ Department of Paediatrics, Division of Paediatric Oncology All India Institute of Medical Sciences New Delhi India; ^5^ Department of Paediatrics Vardhman Mahavir Medical College and Safdarjung Hospital New Delhi India; ^6^ Max Super‐Speciality Hospital, Medical Oncology New Delhi India

**Keywords:** abandonment of treatment, childhood cancer, India, patient tracking, social support

## Abstract

**Background:**

Abandonment of treatment, a major cause of treatment failure in low‐ and middle‐income countries like India, is particularly high during the diagnostic and initial phase of treatment. Tracking of patients during this risk period may reduce treatment abandonment rates and increase quality of care.

**Aim:**

The primary aim was to pilot the use and check the acceptability of a tool for tracking children with cancer in New Delhi during the initial part of their treatment. Secondary aim was to estimate abandonment rates among these patients.

**Methods:**

This prospective study was carried out in two centers of North India in New Delhi and enrolled children less than 18 years diagnosed with cancer at these centers and who had registered with Cankids for social support. Parent support group (PSG) workers maintained contact with the child's family at least once a week for the first 12 weeks. Details of each contact and subsequent action were recorded in a customized book (called “You are not alone” or YANA Book). Descriptive analysis of these contacts was done in Microsoft Excel and presented in frequencies and percentages. The five‐point Likert scale was used to check the acceptability of the tool among the PSG workers.

**Results:**

Seven PSG workers enrolled and tracked 81 patients (73% male with a median age of 6 years). During the 12‐week study period, 986 contacts were attempted and three (3.7%) patients had abandoned their treatment. All PSG workers strongly agreed that the YANA book was simple to understand and use, decreased their workload, and helped provide better assistance to patients.

**Conclusion:**

The tool for patient tracking was well accepted by the PSG workers and considered easy to use. We now plan to implement our model as a routine service at all the partnering hospitals in India.

## 1 INTRODUCTION

In recent years, there has been a significant advancement in the treatment modalities of childhood cancer and consequently in the outcome of childhood cancer. But in low‐ and middle‐income countries (LMICs) like India, treatment abandonment remains high and is one of the major causes of treatment failure with a reported rate of 10% to 62%.^1^, ^2^, ^3^, ^4^ Treatment abandonment is defined as the failure to start or complete therapy for a disease that could be cured or controlled, and missing treatment for a period of four or more weeks from the scheduled treatment, without a medically indicated cause.^1^

The financial situation and poor economic status of the families are among the leading causes of treatment abandonment in LMICs. However, several other factors impact treatment abandonment. These include gender, maternal literacy, refusal of mutilating surgeries (amputation and enucleation when indicated), difficulties coping with the impact of the disease, difficulty in understanding medical instructions, distance to center, and parents with social vulnerabilities.^5^, ^6^ Treatment abandonment is particularly high during the diagnostic and initial phase of treatment. In a study conducted on children with acute lymphoblastic leukemia, 64% of patients had abandoned treatment during the diagnostic evaluation or induction phase of treatment.^7^

Understanding the factors contributing to treatment abandonment allows for the development of appropriate interventions. Although the published literature on interventions is relatively sparse compared to the information on the burden and factors associated, we do know that one or more interventions when applied singly or in combination can have a dramatic reduction in abandonment.^2^, ^8^ Counseling of patients and caregivers and providing social support have shown to lead to a reduction of treatment abandonment rates in India.^9^, ^10^Patient tracking is one potential area of intervention worthy of investigation. A study on 28 patients with retinoblastoma demonstrated improvement in compliance when parents were reminded telephonically about scheduled appointments.^11^

Cankids Kidscan (www.cankidsindia.org) is a grassroots organization working for the change for childhood cancer in India and its primary activity is providing support to children with cancer and their families throughout the journey of their cancer diagnosis and treatment. It was set‐up in the year 2004 under the aegis of Indian Cancer Society (Delhi) and then registered as an independent national society in the year 2012. It has a partnership with 104 hospitals across 22 states and 47 cities of India. Through partnerships with individual cancer centers, it enables better standards of treatment and care by assessing needs and providing necessary social support through funds and facilitation for medication, investigations and supportive care.

We hypothesized that developing a systematic strategy to track all the children with cancer who registered with Cankids in the initial part of their treatment journey combined with ongoing social support which was already being offered to the families would reduce abandonment rates. To conduct such a study, we developed the tools for tracking to make the capture of relevant information easier for our frontline workers (parent support group workers or the PSG workers) and maintain uniformity in data recording. Cankids employs 59 59 PSG workers across India. These PSG workers are the parents and/or the family members of the children with cancer who have experienced the cancer journey and then are trained to offer emotional support, counseling, navigation, education, and awareness to other families. As part of their role, they routinely act as a bridge between the healthcare providers and patient for cancer‐directed treatment. If any relevant medical or social issue is flagged up, it is communicated to the relevant healthcare professionals as appropriate.

Our primary objective was to pilot the use and check the acceptability of the tool for tracking children with cancer in New Delhi during the initial part of their treatment. The secondary objective was to estimate abandonment rates among these patients.

## 2 METHODS

### 2.1 Study setting and patient eligibility

This prospective study was carried out in North India in the capital New Delhi. Eligibility criteria for inclusion were newly diagnosed children (<18 years age) with cancer who commenced their treatment at one of the two large public sector hospitals. All India Institute of Medical Sciencesand Safdarjung Hospital and had subsequently registered with Cankids for social support. They were approached to be enroled in the study. These two study centers are among the busiest public sector hospitals in North India and serve as major referral units for other hospitals across the country (in particular North, Central, and East India). The All India Institute of Medical Sciences has an annual newly diagnosed childhood cancer burden of around 1500 and Safdarjung Hospital of around 300. They and the majority of their patients are from the lower socioeconomic strata of society. These hospitals work with local organizations like Cankids to help provide accomodation wherever needed. Abandonment rates range from to 20% to 25% based on the limited information available for retinoblastoma patients.^4^, ^11^

Patient recruitment for the study was done for a period of 3 months from September to November 2014. The consent was taken by the PSG worker at the time of registration to seek social support for every enrolled child diagnosed with cancer.

### 2.2 Developing the patient tracking tool and procedures

Before this study, patient tracking at Cankids used to be done, but there was no specific method to the process and documentation (eg, handwritten notes in diaries may or may not have been recorded). This used to result in missing data and inconsistent implementation of patient tracking. To structure the tracking process and to ensure an adequate and uniform action on each follow‐up, a specific record book called the “You are not alone” or YANA Book was created as shown in Appendix. Each PSG Worker was given one such book which they used to track all the patients assigned to them.

**FIGURE 1:**
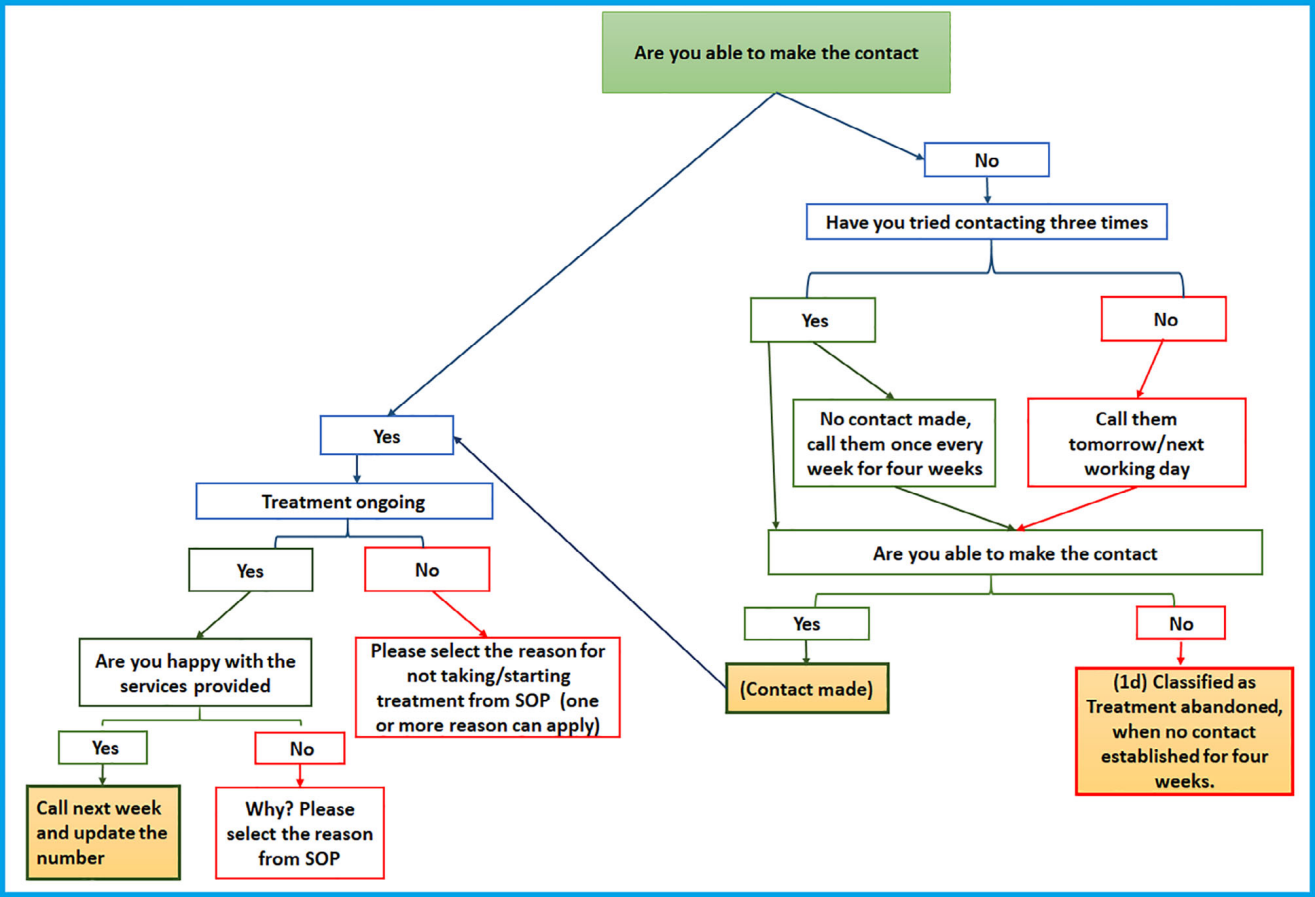
Weekly follow‐up process

### 2.3 The process for follow‐up

The PSG worker made the initial contact with the child and child's family and then maintained the contact with the family for every week for 12 successive weeks either by phone or in‐person through the weekly follow‐up process as shown in Figure 1. For each contact attempted, there were three outcomes color‐coded to resemble the traffic light system (Appendix) ‐ contact not made; contact made and treatment not ongoing; contact made and treatment ongoing. An action plan was developed for each of these outcomes (Appendix). The possible responses were prefilled, and the PSG worker had to tick or circle the appropriate response. There was a section to add comments.

### 2.4 Data analysis and statistics

The data collected through the YANA booklet were transferred to Microsoft Excel for further analysis. Descriptive analysis of collected data was done and presented in frequencies and percentages. The five‐point Likert scale was used to check the acceptability of the tool among the PSG workers.

## 3 RESULTS

### 3.1 Patient cohort

A total of 81 (73% male with a median age of 6 years) patients were enrolled and tracked by seven PSG workers between September to November 2014. The children enrolled were diagnosed with acute leukemia (31%), retinoblastoma (27%), bone sarcomas (15%), lymphoma (11%), and others (16%). In total, 78% of the patients were from outside the Delhi National Capital Region (including one patient who was from outside India).

### 3.2 Patient tracking

A total of 986 contacts (62% on phone or 38% in‐person) during 12 weeks of follow‐up were attempted. Totally, 92% (n = 893) of total contacts were successful at first attempts, while 9% (n = 92) contact could not establish at the first attempt. At the end of 12‐week tracking, 70 patients were alive and were on treatment, six (7.4%) children died, and three (3.7%) had abandoned their treatment, whereas two (2.5%) were not offered further curative treatment. Those who abandoned including the child from Afghanistan did not offer any reason.

### 3.3 Tool acceptability

**FIGURE 2:**
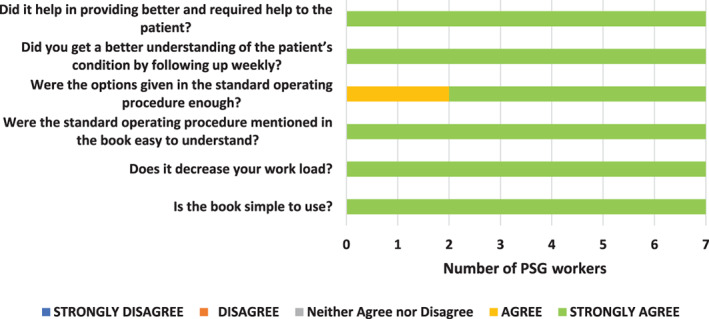
Result on the acceptability of tool (five‐point Likert scale)

As shown in Figure 2, all PSG workers strongly agreed that the YANA book was simple to understand and use, decreased their workload, and helped provide better assistance to patients.

## 4 DISCUSSION

Our study has demonstrated that the tool developed for patient tracking was well accepted and considered simple to execute by all PSG workers. It decreased the workload and made it easy to track patients. At the end of the 12‐week tracking, 3.7% of patients had abandoned treatment. As we recognize that most treatment abandonment happens initially,^12^, ^13^ we would expect that the eventual abandonment rate would be 4% to 5%. As there was no control cohort (and the study was not designed to answer the question), we cannot quantify the impact of tracking on abandonment in our study, but this compares favorably with the limited historical data from these institutes.^4^, ^11^ More recent experience from Tata Memorial Hospital demonstrates that abandonment rates of childhood cancer patients treated in public sector hospitals in India can be reduced to less than 5% when patients are prospectively tracked along with providing holistic support.^14^

At the time of conduct of this study, there was no published literature on the role of patient tracking or the tools in the context of childhood cancer treatment and its benefits. Subsequent to our work, four studies have shown the development, implementation, and impact of such a models. The first experience was from Benjamin Bloom Hospital in El Salvador.^15^ Families of all childhood cancer patients who missed scheduled appointment were contacted and interviewed to ascertain and address the reasons with an escalation plan in case of failure to contact or return. Of the 25 953 appointments scheduled during January 2011 to December 2012, 1111 appointments were missed, and these patients were tracked and consequently. Consequently, they recorded a decline in the annual rate of abandonment from 13% to 3%. Another study by Ferman et al on childhood cancer patients, at The Brazilian National Cancer Institute, Brazil, during January 2012 to December 2017 showed that patient tracking after missed appointments in combination with increased social support for some of these patients led to a reduction in abandonment rate 0.9%.^16^ Similar experience has also emerged from Paraguay where tracking with community‐based interventions for missed appointments has led to a significant fall in abandonment rates in countries from 20% to 0%.^17^ In India, the 11 011 children with cancer, who missed their appointments at Tata Memorial Hospital in Mumbai from 2009 to 2016, were prospectively tracked along with providing holistic support (counseling by the dedicated social support team, accommodation, food, etc.). They reported a reduction in the rate of abandonment from 20% to 3.7%.^14^

In each of these studies, social support was already ongoing or started along with tracking. This is similar to what we have done in our cohort with social support being provided by Cankids. Our model, however, had one crucial difference from those described above. The trigger for tracking was not a missed scheduled appointment, but even earlier from the start of treatment regardless of whether an appointment was scheduled or not. This was because we recognize that the highest risk of abandonment is during the initial phase of treatment. While Cankids is a social support organization complimenting and working in conjunction with the health professionals in these hospitals, it does not have access to the appointment scheduling of patients and hence cannot keep track of missed appointments. So tracking all patients every week regardless of their schedule was a simple and feasible solution. This further has helped to do early counseling of caregivers to make them understand the importance of adherence to their child's treatment and provide the best possible social support. It would be better and preferable if such a model can be implemented in collaboration with the hospital, as it would reduce duplication of efforts and be more effective.

A limitation of this study is that it was conducted in the year 2014. Despite this, we believe, this study still remains relevant because the problem of treatment abandonment is still a major concern,^18^ the tool which we developed is applicable even now and the model centered around services by a social support organization has previously not been reported.

We now plan to implement our model as a routine service at all the partnering hospitals in India where Cankids is providing social support. Also, we are looking to work with hospitals and doctors to develop a combined strategy to implement this model for optimum outcomes.

## AUTHOR CONTRIBUTIONS

**Shivani Ahuja:** Conceptualization; data curation; formal analysis; investigation; project administration. **Jyotsna Sharma:** Formal analysis; writing‐original draft; writing‐review and editing. **Sumit Gupta:** Conceptualization; writing‐review and editing. **Sameer Bakhshi:** Data curation; writing‐review and editing. **Rachna Seth:** Data curation; writing‐review and editing. **Amitabh Singh:** Data curation; writing‐review and editing. **Poonam Bagai:** Conceptualization; project administration; resources; writing‐review and editing. **Ramandeep Arora:** Conceptualization; data curation; formal analysis; methodology; project administration; supervision; writing‐original draft; writing‐review and editing.

## CONFLICT OF INTEREST

The authors report no conflicts of interest. The authors alone are responsible for the content and writing of the article.

## ETHICS STATEMENT

Mandatory ethics approval was obtained from an independent ethics committee.

## Supporting information


**Appendix S1.** Supporting Information.

## Data Availability

The data that support the findings of this study are available on request from the corresponding author.
