# Supplementary material for: Patient tracking during treatment of children with cancer in India – An exploratory study
Source: Cancer Rep (Hoboken). 2021 Feb 23;5(6):e1359. doi: 10.1002/cnr2.1359 (PMC9199505; doi:10.1002/cnr2.1359)

# Appendix

The YANA Book Cover Page


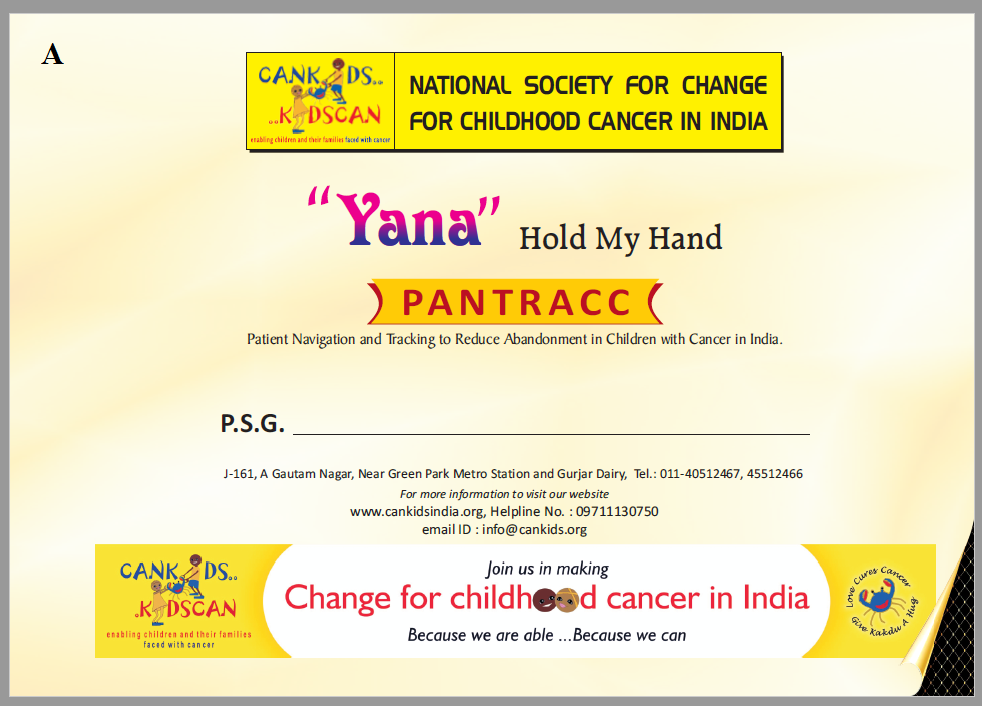


The YANA Book opening summary page to record each child’s ID, name, diagnosis, date of diagnosis, date of first meeting/contact with the patient by PSG workers, and the page number where all successive contact for 12 weeks were recorded.
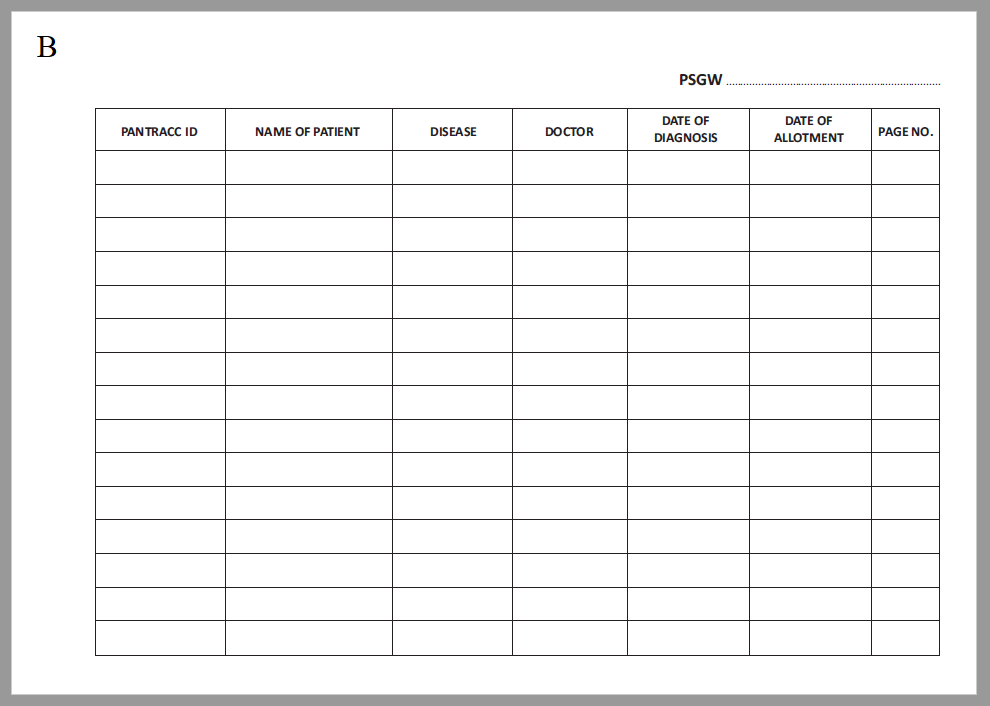


The YANA Book individual pages for each child with additional patient details and the log of each contact made and its outcome.


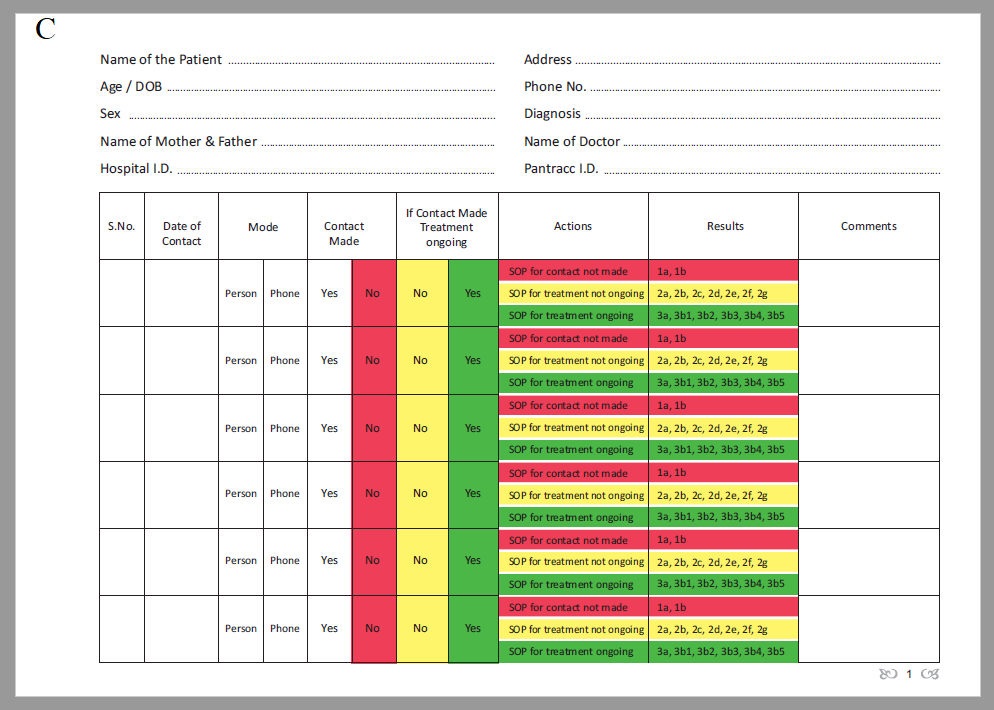


The YANA Book procedures to be followed depending on the response given.
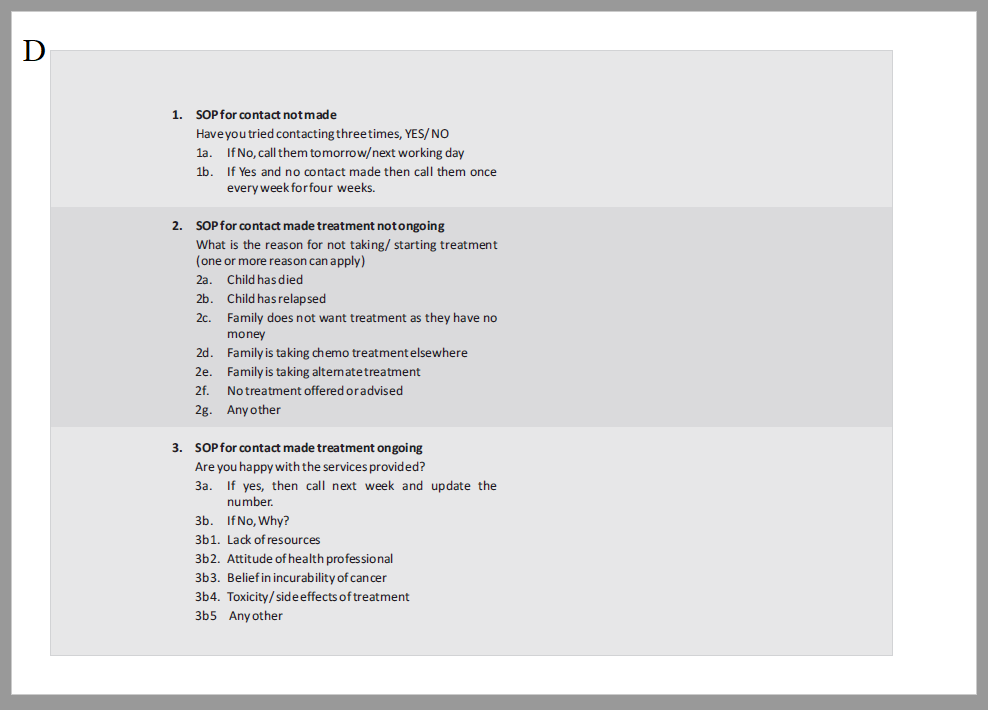

Supplement: Supplementary file 1 — Appendix S1. Supporting Information. [file CNR2-5-e1359-s001.docx]
